# PPP1R12A Copy Number Is Associated with Clinical Outcomes of Stage III CRC Receiving Oxaliplatin-Based Chemotherapy

**DOI:** 10.1155/2015/417184

**Published:** 2015-05-31

**Authors:** Chenbo Zhang, Ajian Li, Huaguang Li, Kangsheng Peng, Qing Wei, Moubin Lin, Zhanju Liu, Lu Yin, Jianwen Li

**Affiliations:** ^1^Department of General Surgery, Ruijin Hospital, Shanghai Jiaotong University School of Medicine, Shanghai 200025, China; ^2^Center for Translational Medicine, Yangpu Hospital Affiliated to Shanghai Tongji University School of Medicine, Shanghai 200090, China; ^3^Department of Gastroenterology, The Tenth People's Hospital of Tongji University, Shanghai 200072, China; ^4^Department of Pathology, The Tenth People's Hospital of Tongji University, Shanghai 200072, China; ^5^Department of General Surgery, Yangpu Hospital Affiliated to Shanghai Tongji University School of Medicine, Shanghai 200090, China

## Abstract

*Aim*. To investigate the correlation between PPP1R12A gene copy number and clinical outcomes of oxaliplatin-based regimen in stage III colorectal cancer (CRC). *Methods*. A total of 139 paraffin-embedded tissue samples of stage III CRC patients who received oxaliplatin-based treatment after radical surgery were recruited. Genomic DNA was extracted and purified from paraffin-embedded sections. Quantitative PCR methods were used to detect the relative copy number (RCN) of PPP1R12A. *Results*. Statistical analysis demonstrated that low PPP1R12A RCN was associated with poor RFS (HR = 2.186, 95% CI: 1.293–3.696; *P* = 0.003) and OS (HR = 2.782, 95% CI: 1.531–5.052; *P* < 0.001). Additionally, when patients were stratified according to subgroups of stage III and tumor location, poor RFS and OS were also observed in the low PPP1R12A RCN group with significance (RFS: IIIB HR = 2.870, *P* < 0.001; colon HR = 1.910, *P* = 0.037; OS: IIIB HR = 3.527, *P* < 0.001; IIIC HR = 2.662, *P* = 0.049; rectum HR = 4.229, *P* = 0.002). *Conclusion*. Our findings suggest the copy number of PPP1R12A can independently predict recurrence and overall survival of stage III colorectal cancer patients receiving oxaliplatin-based chemotherapy.

## 1. Introduction

Colorectal cancer (CRC) is the third most commonly diagnosed cancer in males and the second in females worldwide [1, 2] and the incidence rate and mortality rate attributed to CRC have been increasing in recent years in China. For the treatment of advanced CRC, chemotherapy is recommended and oxaliplatin-based regimens remain the first line chemotherapy in colorectal cancer. The recurrence-free survival of advanced CRC patients has been improved since the introduction of oxaliplatin-based chemotherapy treatment [3]. However, the chemoresistance remains a major obstacle and the treatment efficacy has reached a plateau [4, 5]. Therefore, it is urgent to identify novel biomarkers capable of predicting the efficacy of oxaliplatin-based treatment [6].

PPP1R12A (protein phosphatase 1 regulatory subunit 12A) belongs to the myosin phosphatase targeting protein (MYPT) family and is also known as myosin phosphatase target subunit 1 (MYPT1). PPP1R12A molecule possesses a PP1c-binding motif, via which PPP1R12A recognizes and binds PP1c*δ* (protein phosphatase 1, catalytic subunit, *δ* isoform) to form the PP1c*δ*-MYPT1 complex. Through modulating the catalytic activity and specificity of PP1c*δ*, PPP1R12A has been reported to participate in diverse cellular functions, such as smooth muscle contraction [7–9], cell migration and adhesion [10, 11], cell cycle regulation [12–14], and embryonic development regulation [15]. In addition, PPP1R12A possibly plays an important role in cancer chemoresistance and prognosis. Merlin, a tumor suppressor, is activated when the serine 518 is dephosphorylated by the PPP1R12A–PP1c*δ* [16]. Merlin loss leads to a coordinated increase of Wnt/*β*-catenin signaling [17] and PI3K/AKT pathway [18], both of which promote chemoresistance in colorectal cancer [19, 20]. Thus, we hypothesize that PPP1R12A associates with the recurrence and overall survival in colorectal cancer. However, so far there have been no studies exploring the correlation between PPP1R12A and clinical outcomes in colorectal cancer.

In this study, we employed the quantitative PCR to detect the relative copy number (RCN) of PPP1R12A in stage III CRC patients receiving oxaliplatin-based regimen and compute the association of PPP1R12A gene copy number with clinical outcomes.

## 2. Materials and Methods

### 2.1. Human Subjects

This study included a total of 139 paraffin-embedded tissue samples of the patients who received curative resection from 2006 to 2011 in the Ruijin Hospital affiliated to Shanghai Jiao Tong University and the Tenth People's Hospital affiliated to Shanghai Tongji University. All the patients were histologically and clinically diagnosed as the stage III of CRC according to the criteria proposed by the Standard American Joint Committee on Cancer (AJCC) and only patients receiving at least 6 cycles of oxaliplatin-based chemotherapy after operation were included in this study. None of the patients received chemotherapy or radiotherapy before radical surgery. Written informed consent was obtained from all participants. This study was approved by the Medical Ethical Committee of Shanghai Jiaotong University and Tongji University.

### 2.2. DNA Extraction

Genomic DNA was extracted and purified from 5 *μ*m thick formalin-fixed paraffin-embedded sections (tumor cells account for at least 70%) of the 139 tumors using a DNeasy kit (Qiagen, CA, USA) according to the manufacturer's instructions.

### 2.3. Real-Time Quantitative PCR

To detect PPP1R12A gene DNA copy number, real-time quantitative polymerase chain reaction (qPCR) was performed using IQ5 Multicolour Real-Time PCR Detection system (Bio-Rad, CA, USA). qPCR was carried out using the SYBR Green kit (Qiagen, Germany) in a final volume of 20 *μ*L with housekeeping gene glyceraldehyde 3-phosphate dehydrogenase (GAPDH) used as an internal control. The primers for GAPDH were forward, 5′-ACG AAT TTG GCT ACA GCA ACA GG-3′, and reverse, 5′-CCA GCA GTG AGG GTC TCT CTC TT-3′. The primers for PPP1R12A were forward, 5′-AGG TGA AGT TCG ACG ATG GC-3′, and reverse, 5′-TCC GTC CAC ATT GGC GTA AT-3′. Each thermal cycle consisted of 15 s of denaturation at 95°C, 20 s of primer annealing at 60°C, and 20 s of extension at 72°C. All the samples (both PPP1R12A and GAPDH) were run in triplicate.

### 2.4. Statistical Analysis

Each assay was tested in triplicate. Data points that generated triplicate Ct values with over one cycle variance were excluded from analysis. Since the samples were analyzed in triplicate, values for each triplicate sample were averaged to the final data use. The mean Ct value obtained from each sample was normalized to the averaged copy number and then subjected to analysis with the 2^−ΔΔCt^ method [21, 22].

We performed the Pearson Chi-square (*χ* ^2^) to compare the distributions of categorical variables. Recurrence-free survival (RFS) was calculated from the surgery date to the date of disease recurrence or death. Overall survival (OS) was defined as the time from the date of surgery to that of death or the end of follow-up. We dichotomized patients into high RCN and low RCN groups at median, tertile and quartile cut-off points, and it showed that the most significant associations of PPP1R12A RCN with RFS and OS were achieved at first tertile point (Supplemental Tables  1 and  2, in Supplementary Material available online at http://dx.doi.org/10.1155/2015/417184). So we chose the first tertile of PPP1R12A copy number as the cut-off point in our study [23]. Hazard ratios (HRs) and 95% confidence intervals (CIs) were estimated using Kaplan-Meier analysis, as well as multivariate COX regression, while adjusting for age, location, and gender. All statistical tests were two-sided. Statistical analysis was performed using the SPSS Software, version 20.0 (Chicago, IL, United States). *P* < 0.05 was considered statistically significant.

## 3. Result

### 3.1. Patient Characteristics

The demographic and clinical characteristics in the 139 patients with CRCs were summarized in Table 1. The patient pool comprised eighty-five (61.2%) males and fifty-four (38.8%) females. Of the 139 patients, the median age was 61 years (range, 25–84) with the mean age of 61.06 years. The tumors were found in the colon (*n* = 72, 51.8%) and rectum (*n* = 67, 48.2%). The patients with stage IIIA, IIIB, and IIIC disease accounted for 9.4% (*n* = 13), 71.2% (*n* = 99), and 19.4% (*n* = 27), respectively. After a median follow-up of 49 months (range, 4–98 months), 56 patients (40.3%) exhibited tumor recurrence and 44 patients (31.7%) died.

### 3.2. Correlation between PPP1R12A Copy Number and Recurrence-Free Survival

**Figure 1:**
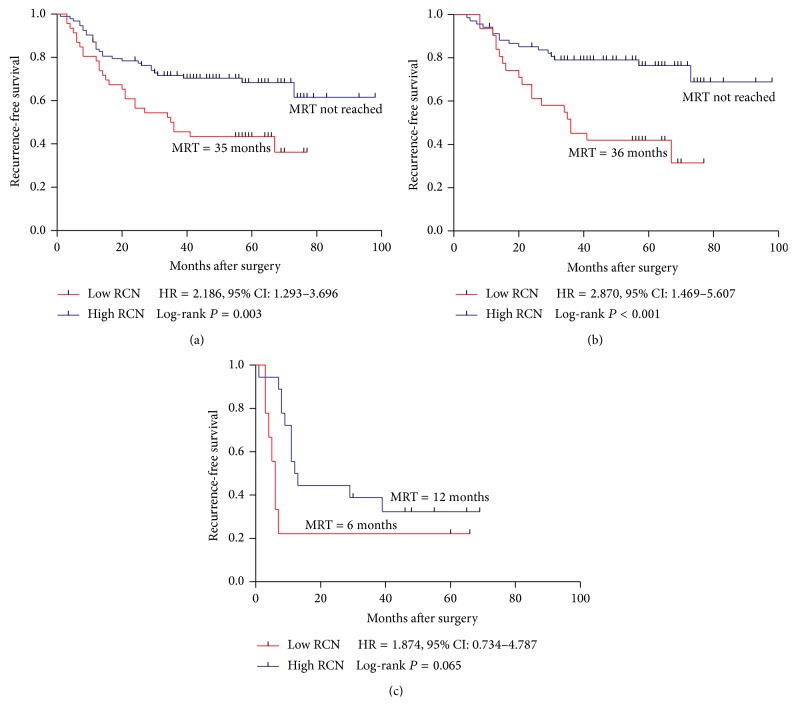
Kaplan-Meier survival curves of patients with stage III, IIIB, and IIIC disease receiving oxaliplatin-based chemotherapy. (a) RFS in total stage III; (b) RFS in stage IIIB; (c) RFS in stage IIIC. MRT: median recurrence time.

**Figure 2:**
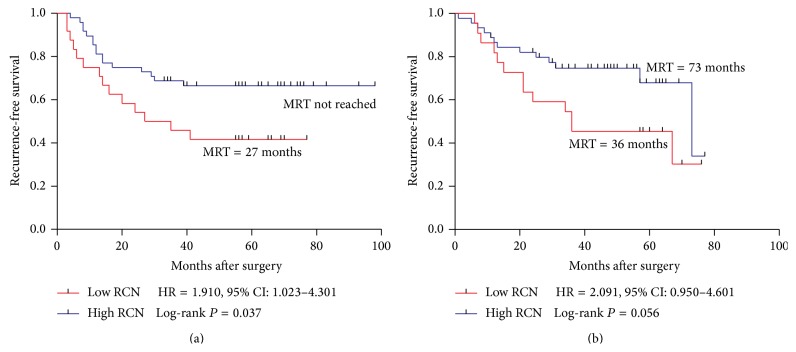
Kaplan-Meier survival curves of patients with stage III, IIIB, and IIIC disease receiving oxaliplatin-based chemotherapy. (a) RFS in colon; (b) RFS in rectum. MRT: median recurrence time.

Patients were dichotomized into high RCN (RCN ≥ 0.37, *n* = 93) and low RCN (RCN < 0.37, *n* = 46) groups with a tertile cut-off of PPP1R12A relative copy number (RCN = 0.37). During the follow-up, 56 (40.3%) of the 139 patients suffered recurrence (Table 1). The median recurrence-free periods were 35 (±9.6) months in the low RCN group while the median RFS were not reached in the high RCN group (Figure 1(a)). Both the univariate analysis and the multivariate analysis of RFS were shown in Table 2. High lymph node metastasis (lymph  node ≥ 4: HR = 2.160, *P* = 0.007), high TNM stage (IIIC: HR = 4.001, *P* < 0.001), and low RCN of PPP1R12A (RCN < 0.37: HR = 2.186, *P* = 0.003) were significantly associated with poor RFS. After adjusting for age, location, gender, TNM, lymph node metastasis, and histology grade, PPP1R12A remained an independent factor associated with RFS. In the Kaplan-Meier analysis, the curves showed that patients with low PPP1R12A copy number were significantly associated with poor RFS (*P* = 0.003) (Figure 1(a)). To investigate the correlation between PPP1R12A and RFS in subgroups of patients, we performed RFS analysis among IIIB, IIIC, colon cancer, and rectal cancer, respectively. The small size of IIIA patients remained the limitation towards further analysis. Patients with low RCN of PPP1R12A showed poorer RFS than high RCN patients with high significance in IIIB (HR = 2.870, *P* < 0.001) (Figure 1(b)). Also, low RCN of PPP1R12A was associated with poor RFS in colon cancer with significance (*P* = 0.037) (Figure 2(a)). In the IIIC group and rectum group, patients with low PPP1R12A RCN showed a trend toward poor RFS (IIIC: HR = 1.874, *P* = 0.065, rectum HR = 2.091, *P* = 0.056) (Figures 1(c) and 2(b)).

### 3.3. Correlation between PPP1R12A Copy Number and Overall Survival

**Figure 3:**
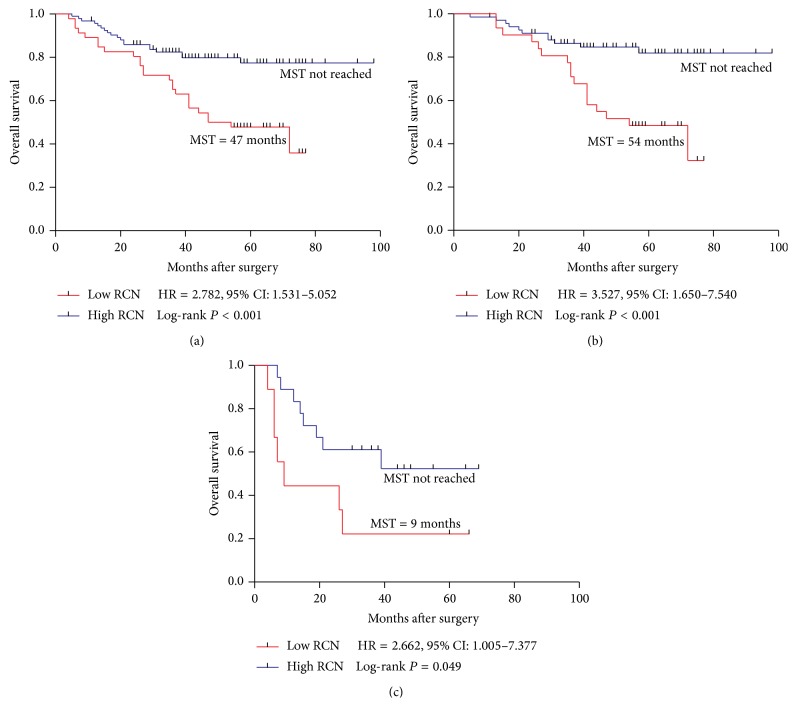
Kaplan-Meier survival curves of patients with stage III, IIIB, and IIIC disease receiving oxaliplatin-based chemotherapy. (a) OS in total stage III; (b) OS in stage IIIB; (c) OS in stage IIIC. MST: median survival time.

**Figure 4:**
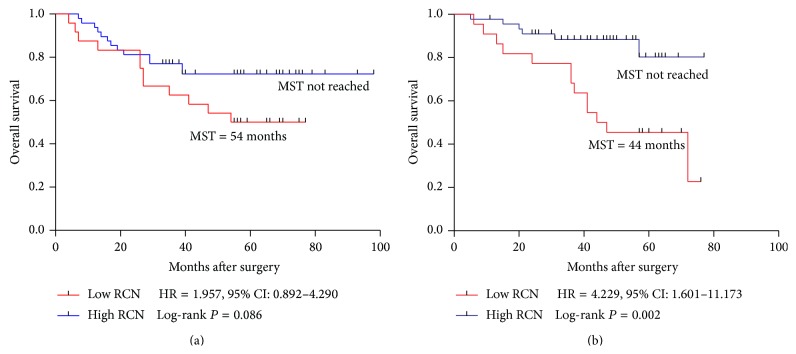
Kaplan-Meier survival curves of patients with stage III, IIIB, and IIIC disease receiving oxaliplatin-based chemotherapy. (a) OS in colon; (b) OS in rectum. MST: median survival time.

Among the 139 stage III CRC patients, 44 (31.7%) patients died during the follow-up. The median overall periods were 47 (±11.1) months in the low RCN group while the OS were not reached in the high gene copy number group (Figure 3(a)). Both the univariate analysis and the multivariate analysis of OS were shown in Table 3. Poor differentiation (HR = 2.310, *P* = 0.011), high lymph node metastasis (lymph  node ≥ 4: HR = 2.750, *P* = 0.001), high TNM stage (IIIC: HR = 3.613, *P* < 0.001), and low PPP1R12A RCN (RCN < 0.37: HR = 2.782, *P* < 0.001) were shown to be significantly associated with poor OS. Furthermore, the multivariate analysis showed that poor differentiation (HR = 2.242, *P* = 0.021), TNM stage (IIIC: HR = 3.913, *P* < 0.001), and low PPP1R12A (HR = 2.976, *P* = 0.001) remained independent predictive factors of poor OS. The Kaplan-Meier analysis was also used to assess the correlation between PPP1R12A copy number and overall survival. The log-rank test showed that patients with low PPP1R12A copy number were associated with a statistically significant poor OS (*P* < 0.001) (Figure 3(a)). Further analysis in different subgroups showed low RCN was still associated with OS in IIIB (IIIB: *P* < 0.001, HR = 3.527) and IIIC (IIIC: *P* = 0.049, HR = 2.662) patients (Figures 3(b) and 3(c)). In the rectum subgroup, low RCN was significantly correlated with poor OS (*P* = 0.002, HR = 4.229) (Figure 4(b)). Similarly, patients with low PPP1R12A RCN showed a trend towards poor OS in colon subgroup (Figure 4(a) *P* = 0.086, HR = 1.957).

## 4. Discussion

Colorectal cancer remains one of the most prevalent cancer and leading causes of cancer deaths worldwide. Adjuvant chemotherapy is thus required to reduce relapse rate and enhance survival. Oxaliplatin-based regimen is widely used as first-line therapy for advanced colorectal cancer; however, the recurrence is still high. So identification of patients who will likely or unlikely benefit from chemotherapy would allow personalized treatment of CRC patients.

Here for the first time we conducted the study to examine the possibility of PPP1R12A as a possible biomarker for the prediction of clinical outcomes in stage III colorectal cancer patients treated with oxaliplatin-based chemotherapy. The results showed that patients with low copy number of PPP1R12A had a poor OS and RFS with statistical significance, indicating PPP1R12A was able to predict recurrence and overall survival. PPP1R12A is essential in the activation of Merlin [16]. Serine 518 of Merlin is dephosphorylated by phosphatase (PPP1R12A–PP1c). And the dephosphorylated merlin exerts inhibitory effect on downstream signaling pathways, for example, PI3K/Akt and Wnt/*β*-catenin signaling [17, 18]. Recent studies have demonstrated that inhibition of PI3K would lead to increased oxaliplatin-induced cell apoptosis and increased oxaliplatin sensitivity in cholangiocarcinoma cells [24]. In addition, Wnt/*β*-catenin pathway has been revealed to enhance chemoresistance in many cancer cell types including hepatocellular carcinoma and neuroblastoma and this kind of chemoresistance is thought to be associated with overexpression of multidrug resistance 1 gene (MDR1) [25, 26]. Probably PPP1R12A activates Merlin which represses PI3K/Akt and Wnt/*β*-catenin signaling and leads to inhibition of drug resistance and enhances the apoptosis induced by oxaliplatin.

Conventional TNM staging system is not able to predict the risk of recurrence among certain subgroup patients such as IIIB and IIIC. In this study, we showed that PPP1R12A RCN was significantly correlated with OS and RFS even in IIIB and IIIC. This indicates PPP1R12A can not only predict OS and RFS of stage III CRC patients, but also predict the OS and RFS of IIIB and IIIC patients. This compensates the weakness of TNM staging and allows clinicians to make the more accurate therapeutic decision.

In conclusion, our study demonstrates that the copy number of PPP1R12A could independently predict recurrence and overall survival of stage III colorectal cancer patients receiving oxaliplatin-based adjuvant chemotherapy.

## Supplementary Material

Supplementary Material: Median, tertile and quartile points of the PPP1R12A RCN distribution were chosen to dichotomize patients into high RCN and low RCN groups. The associations of PPP1R12A RCN with RFS and OS at these cut-offs were analyzed and the results were shown in supplemental table 1 and 2.

## Figures and Tables

**Table 1:** Selected demographic and clinical characteristics of patients with CRCs.

Clinicopathological factors	Number of patients	Recurrence	*P* value	Dead	*P* value
	*N* = 139	Number (%)		Number (%)	
Age (years)		56		44	
≥65	53 (38.1%)	22/53 (41.5%)	0.818	19/53 (35.8%)	0.404
<65	86 (61.9%)	34/86 (39.5%)		25/86 (29.1%)	
Sex					
Male	85 (61.2%)	33/85 (38.8%)	0.659	25/85 (29.4%)	0.476
Female	54 (38.8%)	23/54 (42.6%)		19/54 (35.2%)	
Tumor site					
Rectum	67 (48.2%)	26/67 (38.8%)	0.731	19/67 (28.4%)	0.42
Colon	72 (51.8%)	30/72 (41.7%)		25/72 (34.7%)	
Histology					
Well/moderate	113 (81.3%)	43/113 (38.1%)	0.263	31/113 (27.4%)	**0.026**
Poor	26 (18.7%)	13/26 (50.0%)		13/26 (50.0%)	
Lymph node metastasis					
≥4	31 (22.3%)	18/31 (58.1%)	**0.022**	17/31 (54.8%)	**0.002**
<4	108 (77.7%)	38/108 (35.2%)		27/108 (25.0%)	
TNM stage					
IIIA	13 (9.4%)	2/13 (15.4%)	**0.001**	1/13 (7.7%)	**0.004**
IIIB	99 (71.2%)	35/99 (35.4%)		28/99 (28.3%)	
IIIC	27 (19.4%)	19/27 (70.4%)		15/27 (55.6%)	

Bold items highlight *P* < 0.05; TNM: tumor node metastasis.

**Table 2:** Univariate and multivariate analyses for recurrence-free survival (Cox proportional hazard model).

Variable	Univariate analysis			Multivariate analysis		
	HR	95% CI	*P* value	HR	95% CI	*P* value
Age (≥65 versus <65 year old)	1.116	0.652–1.908	0.689			
Sex (male versus female)	0.844	0.495–1.441	0.535			
Tumor site						
Colon versus rectum	1.075	0.635–1.821	0.788			
Histology						
Poor versus well/moderate	1.548	0.832–2.881	0.168			
Lymph node metastasis						
*n* ≥ 4 versus *n* < 4	2.160	1.230–3.793	**0.007**	0.832	0.409–1.693	0.612
TNM stage						
IIIC versus IIIA + IIIB	4.001	2.281–7.017	**<0.001**	5.057	2.478–10.323	**<0.001**
PPP1R12A (low versus high)	2.186	1.293–3.696	**0.003**	2.596	1.500–4.490	**0.001**

Bold items highlight *P* < 0.05, HR: hazard ratio, CI: confidence interval; TNM: tumor node metastasis.

**Table 3:** Univariate and multivariate analyses for overall survival (Cox proportional hazard model).

Variable	Univariate analysis			Multivariate analysis		
	HR	95% CI	*P* value	HR	95% CI	*P* value
Age (≥65 versus <65 year old)	1.301	0.716–2.363	0.387			
Sex (male versus female)	0.754	0.414–1.372	0.355			
Tumor site						
Colon versus rectum	1.202	0.661–2.187	0.546			
Histology						
Poor versus well/moderate	2.310	1.208–4.418	**0.011**	2.242	1.128–4.455	**0.021**
Lymph node metastasis						
*n* ≥ 4 versus *n* < 4	2.750	1.497–5.050	**0.001**	1.050	0.492–2.243	0.899
TNM stage						
IIIC versus IIIA + IIIB	3.613	1.919–6.803	**<0.001**	3.913	1.831–8.366	**<0.001**
PPP1R12A (low versus high)	2.782	1.531–5.052	**<0.001**	2.976	1.602–5.528	**0.001**

Bold items highlight *P* < 0.05; HR: hazard ratio; CI: confidence interval; TNM: tumor node metastasis.
