# Supplementary material for: PPP1R12A Copy Number Is Associated with Clinical Outcomes of Stage III CRC Receiving Oxaliplatin-Based Chemotherapy
Source: Mediators Inflamm. 2015 May 31;2015:417184. doi: 10.1155/2015/417184 (PMC4465766; doi:10.1155/2015/417184)
Supplement: Supplementary file 1 — Supplementary Material: Median, tertile and quartile points of the PPP1R12A RCN distribution were chosen to dichotomize patients into high RCN and low RCN groups. The associations of PPP1R12A RCN with RFS and OS at these cut-offs were analyzed and the results were shown in supplemental table 1 and 2. [file 417184.f1.docx]

**Supplemental table-1** Univariate analyses for recurrence-free survival at different cutoff points

| cutoff points | HR | 95% CI | p value |
| --- | --- | --- | --- |
| median（RCN=1.16） |  |  |  |
| Low RCN | 1(reference) |  |  |
| High RCN | 0.658 | 0.386-1.121 | 0.124 |
| tertile_33%(RCN=0.37) |  |  |  |
| Low RCN | 1(reference) |  |  |
| High RCN | 0.457 | 0.271-0.774 | **0.003** |
| tertile_67%(RCN=2.36） |  |  |  |
| Low RCN | 1(reference) |  |  |
| High RCN | 1.129 | 0.653-1.951 | 0.665 |
| quartile_25%(RCN=0.29） |  |  |  |
| Low RCN | 1(reference) |  |  |
| High RCN | 0.496 | 0.287-0.859 | **0.012** |
| quartile_75%(RCN=3.30） |  |  |  |
| Low RCN | 1(reference) |  |  |
| High RCN | 0.971 | 0.530-1.778 | 0.923 |

Bold items highlight p<0.05; HR: hazard ratio; CI: confidence interval; RCN: relative copy number

**Supplemental table-2** Univariate analyses for overall survival at different cutoff points

| cutoff points | HR | 95% CI | p value |
| --- | --- | --- | --- |
| median（RCN=1.16） |  |  |  |
| Low RCN | 1(reference) |  |  |
| High RCN | 0.660 | 0.359-1.213 | 0.181 |
| tertile_33%(RCN=0.37) |  |  |  |
| Low RCN | 1(reference) |  |  |
| High RCN | 0.360 | 0.198-0.653 | **0.001** |
| tertile_67%(RCN=2.36） |  |  |  |
| Low RCN | 1(reference) |  |  |
| High RCN | 1.093 | 0.586-2.04 | 0.780 |
| quartile_25%(RCN=0.29） |  |  |  |
| Low RCN | 1(reference) |  |  |
| High RCN | 0.428 | 0.234-0.780 | **0.006** |
| quartile_75%(RCN=3.30） |  |  |  |
| Low RCN | 1(reference) |  |  |
| High RCN | 1.206 | 0.621-2.344 | 0.580 |

Bold items highlight p<0.05; HR: hazard ratio; CI: confidence interval; RCN: relative copy number
